# Joint FAO/IAEA coordinated research project on “use of symbiotic bacteria to reduce mass-rearing costs and increase mating success in selected fruit pests in support of SIT application”

**DOI:** 10.1186/s12866-019-1644-y

**Published:** 2019-12-24

**Authors:** Carlos Cáceres, George Tsiamis, Boaz Yuval, Edouard Jurkevitch, Kostas Bourtzis

**Affiliations:** 1 Insect Pest Control Laboratory, Joint FAO/IAEA Programme of Nuclear Techniques in Food and Agriculture, A-1400 Vienna, Austria; 2 Department of Environmental Engineering, University of Patras, 2 Seferi St., 30131 Agrinio, Greece; 3 Departments of Entomology and Plant Pathology & Microbiology, Faculty of Agriculture, Food and Environment, Hebrew University of Jerusalem, POB 12, 76100 Rehovot, Israel

Insects represent the most abundant and speciose group of animals on this planet having established diverse and rather complex interactions with many prokaryotic and eukaryotic organisms. Although the interactions of insects with plants and vertebrates have been extensively studied, their interactions with microorganisms, despite their major significance from the ecological and evolutionary point of view, are still poorly characterized. However, the importance of the microorganisms on insects’ biology and physiology is gradually being unravelled.

True fruit flies (Diptera: Tephritidae) are no exception, as they have established sophisticated interactions with microorganisms. There are over 5000 species in this family, many of which are destructive pests of fruits and vegetables. Beyond the direct damage caused by flies ovipositing in ripening fruit, some species are highly invasive, and incur major trade problems across and within national and international borders. These result in strict and costly quarantine regulations. The use of insecticides to control these pests, while effective on the short term, is associated with major environmental and health concerns. Accordingly, The Sterile Insect Technique (SIT) is deployed against several fruit fly species. This is an environment-friendly, species-specific and environment-friendly technology which can be used as an additional tool in integrated pest management programmes. As the efficiency of SIT may be hampered by quality control and cost effectiveness, major research efforts have focussed on ways to improve this process. These focus on the quality of the insects reared for release and the reduction of operational costs. The symbiotic relationships of tephritids were recognized as potential targets for improving sterile fly quality and reducing costs of production, and the papers in this collection describe this research effort.

Coordinated Research Projects are a very powerful mechanism to bring together scientists in an attempt to fill knowledge gaps and solve important problems IAEA Member States may be facing. Eight years ago, a coordinated research project (CRP) entitled “Use of Symbiotic Bacteria to Reduce Mass-Rearing Costs and Increase Mating Success in Selected Fruit Pests in Support of SIT Application” was implemented by the Joint FAO/IAEA Division of Nuclear Techniques in Food and Agriculture. Carlos Cáceres of the FAO/IAEA was the coordinator of this initiative which attracted the participation of 21 scientists from 16 countries, originating from diverse scientific fields, with the common goal of disentangling the symbiotic associations between the fruit flies and various microorganisms.

The CRP focused on four key areas of research that could improve the quality management of fruit flies for use in SIT programmes: (1) eliminating the use of expensive ingredients in the larval diet (e.g. brewers / torula yeasts) and chemicals which are used to restrict the presence of unwanted microorganisms thus improving the productivity and quality of mass-reared insect colonies, (2) the use of radiation may disrupt the symbiotic community of mass-reared flies by enabling the presence of some bacterial species while limiting others. Determining the impact of radiation on the symbiotic communities will ultimately result to the development of responses to mitigate these effects in a manner that optimizes SIT efficiency; (3) restoring the symbiotic bacterial community in sterile fruit fly males, before their release, can greatly enhance their mating performance, and (4) it is known that several symbiotic bacteria, such as *Wolbachia*, are able to manipulate mating behaviour of their hosts and/or to induce reproductive alterations which can be exploited for the population control of insect pests and disease vectors. For example, the incompatible insect technique (IIT) is based on the *Wolbachia*-induced phenomenon of cytoplasmic incompatibility, which can be used for the population suppression of a target pest by repeated releases of cytoplasmically incompatible males. In some specific cases, SIT and IIT can be used in combination as complementary control tools.

In this CRP, we seek to extend these approaches to manipulating the diverse microbiota present in SIT targeted insect pests associated to protect fruit and vegetable crops. In addition, four meetings were organized at about 18-month intervals during which the participating scientists reported their findings, exchanged ideas and coordinated their research plans. This special issue comprises the final research results of the CRP (18 research papers) that summarize the outcomes of the research carried out by participants and collaborators during the CRP.

The guest editors are confident that this Cross-Journal Supplement on BMC Microbiology and BMC Biotechnology will provide valuable information regarding the manipulation of insect microbiota in support of sterile insect technique applications against insect fruit pests.

## Data Availability

Not applicable.

